# Nutrition and Exercise Interventions on Skeletal Muscle Physiology, Injury and Recovery: From Mechanisms to Therapy

**DOI:** 10.3390/nu16020293

**Published:** 2024-01-18

**Authors:** Sandro Massao Hirabara, Gabriel Nasri Marzuca-Nassr, Maria Fernanda Cury-Boaventura

**Affiliations:** 1 Interdisciplinary Post-Graduate Program in Health Sciences, Cruzeiro do Sul University, São Paulo 01506-000, Brazil; maria.boaventura@cruzeirodosul.edu.br; 2 Department of Rehabilitation Sciences, Universidad de La Frontera, Temuco 4811230, Chile; gabriel.marzuca@ufrontera.cl

Interventional strategies involving nutrition and physical exercise have been widely proposed to positively modulate skeletal muscle function, in both physiological and pathological states, such as obesity, T2DM, inflammatory diseases, cardiovascular diseases, aging, and sarcopenia [1,2,3]. In this sense, it has been observed that various nutrients or dietary bioactive compounds positively modulate different biological processes, such as metabolism, inflammation, redox balance, mitochondrial function, and gene expression. Several physical exercise protocols have also been proposed to maintain, increase, or recover skeletal muscle function [4,5,6]. Research groups are focusing potential molecular targets to direct further studies to treat and/or prevent skeletal muscle disorders.

Moreover, the role of nutrients associated with physical exercise in organokines (adipokines and myokines) responsible for metabolic health has been investigated. For instance, in obesity, there is an imbalance in the plasma concentration of adipokines, characterized by increased pro-inflammatory adipokines (IL-6, TNF-alpha, leptin, resistin, ANGPTL2, RBB4, asprosin, chemerin, visfatin, CRP6, SPARC, WISP1, and lipocalin-2), which has been proposed to contribute to the development of insulin resistance and decreased levels anti-inflammatory, anti-atherosclerotic and/or insulin sensitivity-increasing adipokines (vaspin, omentin, adiponectin, ZAG, and SFRP5). Strategies for modulating the levels of these adipokines can benefit cardiometabolic health and prevent obesity-related co-morbidities [7]. Most of these adipokines are expressed in response to elevated adiposity and a pro-inflammatory environment [8].

In the original article from Supriya et al. [9] the authors investigated the effect of spirulina supplementation, a dietary supplement extracted from cyanobacteria with potential antioxidant and anti-inflammatory effects (Calella et al., 2022; Behairy et al., 2023), on the adipokine/cytokine and lipid level profile in obese men submitted to a high-intensity exercise training program for 12 weeks. Interestingly, the association of spirulina supplementation with high-intensity exercise training improved the lipid plasma profile (decreased cholesterol, LDL-cholesterol, and triacylglycerides, and raised HDL-cholesterol) and the adipokine/cytokine level profile (reduced CRP, TNF-alpha, MCP-1, IL-6, and IL-8), suggesting that this interventional combination can be a good strategy to improve several risk markers of metabolic and inflammatory diseases associated with obesity. The same group [10], using a high-intensity exercise training protocol (CrossFit) for 12 weeks, also evaluated the combined effect of astaxanthin supplementation, a dietary supplement derived from *Haematoccus pluvialis* algae with several potential beneficial effects on metabolic and inflammatory diseases, such as obesity, T2DM, cardiovascular diseases, metabolic syndrome, and cancer [11]. This association was able to improve the adipokine level profile concomitantly with ameliorations in body composition and lipid profile in obese male individuals. These results corroborate the findings of Saeidi et al. [12] and suggest that this interventional combination can acts as an effective therapy to decrease the deleterious effects of obesity in metabolism and the adipokine and lipid level profile.

Various myokines have been proposed to regulate muscle function and metabolism, including myogenesis, mitochondrial biogenesis, mitophagy, autophagy, satellite cells activation, anti-inflammatory effect, skeletal muscle fat oxidation, and insulin response [13]. Furthermore, some myokines are hypothesized to be the mediators between myocytes and other cells, improving glucose metabolism regulation, lipid oxidation, and cardioprotective function [14]. In the study by Kysel et al. [15], the authors compared two nutritional interventions in healthy men: (1) a diet with cyclical ketogenic reduction and (2) a diet with a nutritionally balanced decrease. During the nutritional interventions, the participants were submitted to both aerobic and resistance exercise training protocols. The level profile of myokines and cytokines was determined pre- and post-intervention. The administration of a diet with a nutritionally balanced reduction increased performance in the endurance test and raised muscle strength associated with osteonectin and musclin levels. However, the diet with ketogenic reduction did not improve endurance performance induced by exercise training, but it reduced the FGF-21 plasma levels. This last factor is secreted by several tissues, such as the liver, skeletal muscle, adipose tissue, and pancreas, regulating metabolism and playing an important role as a result of nutritional reduction or a ketogenic diet [16].

In the original article from Sierra et al. [17], the authors showed the significant effect of adequate energy, sodium, cholesterol, vitamin C, and fiber dietary consumption on exercise-induced myokine production (myostatin, musclin, irisin, BDNF, apelin, IL-15, and FGF-21) in endurance runners, which is important for cardiometabolic adaptations and adequate tissue repair in response to intense physical exercise. The myostatin pathway was also investigated in the work performed by de Carvalho et al. [18]. In this work, the authors determined whether creatine supplementation (monohydrate) associated with a resistance exercise protocol was able to modulate the myostatin signaling pathway, skeletal muscle morphology and the expression of myosin heavy chain isoforms in the soleus muscle (red, predominantly oxidative fibers) and gastrocnemius muscle (white portion, predominantly glycolytic fibers) of Wistar rats. According to their theory, the authors expected that the combination of creatine supplementation and resistance training would promote greater changes in the white portion of the gastrocnemius muscle compared to the soleus muscle by attenuating the expression of the myostatin. Accordingly, their findings followed this proposition—that is, greater hypertrophy and interstitial remodeling in the white gastrocnemius muscle than in the soleus muscle.

Valero-Breton et al. [19] evaluated the effects of two different cycling training programs (eccentric versus concentric exercise protocols) for 12 weeks in patients with chronic obstructive pulmonary disease who were demonstrated to have muscle redox imbalance and reduced daily activities. In this study, several redox parameters and inflammatory biomarkers, as well as cardiometabolic assessments, were investigated. In accordance with the findings, the authors concluded that patients submitted to the concentric exercise protocol presented with increased antioxidant capacity (at rest and exercise-induced), improved insulin sensitivity, and elevated HDL levels compared to the patients who performed the eccentric exercise protocol.

Sarcopenia is a syndrome characterized by decreased muscle mass and strength, resulting in impaired physical performance. Different strategies have been proposed and analyzed with the aim to reverse the effects of sarcopenia. Cuyul-Vásquez et al. [20], through a systematic review with meta-analysis, verified in older people with sarcopenia the effectiveness of resistance exercise training with or without whey protein supplementation on muscle mass and strength, as well as physical performance. Despite some limitations of the analysis, mainly related to the small effect size and the low/very low quality of evidence from the studies performed so far, the authors concluded that resistance exercise training is more effective in increasing skeletal muscle mass and grip strength when associated with whey protein supplementation than when performed without any supplementation or with placebo supplementation. The authors highlighted the relevance of additional studies to completely comprehend this process.

In the study from Barquilha et al. [21], the authors determined the modulating effects of fish oil supplementation, which has been proposed as having anti-inflammatory effects and increasing skeletal muscle function [22,23], on muscle damage markers and inflammatory cytokines after a unique hypertrophic exercise session in healthy eutrophic men trained using a undulating/strength exercise protocol for six weeks. The authors found that the supplementation reduced plasma indicators of muscle injury (reduced plasma CK and LDH activity), inflammation (lowed C-reactive protein and IL-6), and redox balance (elevated GSH:GSSG ratio). The findings of this study suggest that supplementation with fish oil can be an important nutritional intervention for reducing muscle injury, inflammatory processes, and oxidative stress after an intense strength exercise session, mainly in untrained and beginner young adults with an interest in practicing resistance/strength training protocols. The authors highlighted that further works are required to evaluate the effectiveness of this supplementation in other groups, such as trained and older people, as well as the modulating effect of n-3 PUFAs on muscle regeneration. In this sense, the work of Jannas-Vela et al. (2023) [24] explored recent literature data about the potential effects of this supplementation on muscle regeneration in different inflammatory conditions, in particular, the effects of their lipid mediators, including oxylipins and endocannabinoids. Further works are required to determine the modulating effects of n-3 fatty acids on membrane composition and endocannabinoid and oxylipin generation, in association with the regenerative process after muscle damage in physiological and pathological diseases.

Interestingly, in the work from Franceković and Gliemann [25], an overview was provided on the relevance of dietary nutritional compounds, including n-3 PUFA, probiotics, and vitamin D, for endothelial glycocalyx maintenance and preservation. The authors proposed that these nutritional compounds are of particular importance for chronic inflammatory diseases, where endothelial dysfunction and vascular abnormalities are observed, including obesity, T2DM, dyslipidemias, metabolic syndrome, and cardiovascular diseases.

In summary, various groups are joining efforts to understand the molecular targets and cellular systems implicated in the effects of nutritional interventions or different physical exercise protocols on muscle physiology, recovery, and injury in cellular, animal, and human models. Various studies have been published and great advances have been made related to this topic in recent decades. The present Special Issue has further contributed to the advanced comprehension of the molecular targets and cellular systems implicated in skeletal muscle physiology, injury, and recovery in physiological and pathological conditions.
